# Effects of H-Reflex Onset Latency on Gait in Elderly and Hemiplegic Individuals

**DOI:** 10.3390/medicina58060716

**Published:** 2022-05-27

**Authors:** Seon-Chil Kim, Sung-Hyoun Cho

**Affiliations:** 1 Department of Biomedical Engineering, School of Medicine, Keimyung University, 1095 Dalgubeol-daero, Daegu 42601, Korea; chil@kmu.ac.kr; 2 Department of Physical Therapy, Nambu University, 23 Cheomdanjungang-ro, Gwangju 62271, Korea

**Keywords:** assessment, gait, hemiplegia, H-reflex, older adults, stroke, treatment

## Abstract

*Background and Objectives*: The Hoffmann’s reflex (H-reflex) is important in electrodiagnostic testing because it improves sensitivity and specificity in diagnosing radiculopathies. Although quantitative electromyography (EMG) measurements for H-reflex amplitudes during the gait cycle have been performed in both hemiplegic and healthy individuals, research on the H-wave latency in these individuals during the gait cycle is lacking. *Materials and Methods*: The H-reflex latency of the soleus muscle was investigated in hemiplegic stroke patients and healthy elderly persons in this observational analytical study. Two groups of individuals participated in this study: healthy adults (*n* = 25) and stroke patients with hemiplegia (*n* = 25) were compared. An MP150 with Ag-Ag/Cl electrodes was utilized to record and analyse electromyography measurements. All individuals could walk independently indoors. Stimuli were administered to elicit the H-reflex in the four gait phases as the participant walked. *Results*: Stroke patients had a significantly shorter latency than did healthy patients in the mid-swing, mid-stance, and toe-off phases of the gait cycle; heel-strike latency did not significantly differ. *Conclusions*: These results can be used as diagnostic data to help account for patient characteristics or measure the recovery extent for treatment planning and gait training in hemiplegic individuals.

## 1. Introduction

The gait process comprehensively involves both the human nervous and musculoskeletal tissues. It consists of a continuous and repetitious motion that moves the body forward while maintaining stability in the stance phase [1]. Therefore, improving gait ability is an important goal of stroke rehabilitation [2]. The peripheral nervous system chooses the most optimal muscular response based on the information received from the subsystem. Gait controls the body’s alignment and center of gravity on the supporting surface via continuous interactions between the central and peripheral nervous systems. However, lower extremity muscle strength, a lack of mechanical stability, structural problems, or decreased afferent feedback can inhibit gait, which depends on an integrated strategy of feedback and motion [3]. Even a minor biomechanical change in the foot, which is located at the farthest end of the lower extremity movement chain, can affect the posture control strategy [4]. The gait of stroke patients, in particular, is affected by damage to the descending nerve tract and accentuation of the reflex action, which induce asymmetrical function of the lower extremities due to an abnormal synergist activity pattern and coordination dysfunction [5]. A delayed gait cycle and gait speed, temporal bilateral imbalance between the legs, a short stance phase on the affected side, and a longer swing phase characterize the gait pattern of stroke patients. Functional activity is limited by the decreased capacity to produce normal quantities of voluntary muscular contractions [6]. Therefore, in patients with pathological gait, successful treatment requires discovering, objectivizing, quantifying, comparing, and assessing the precise causes and affected sites [7].

It is necessary to evaluate hemiplegic stroke patients objectively and quantitatively to estimate their functional loss and treatment prognosis. Ideally, electrical stimulation can be used to directly or indirectly stimulate the motor cortex and cerebrospinal fluid (CSF) pathway. Subsequent excitation of the stimulated CSF pathway will activate the motor threshold of the peripheral muscles through the respective spinal α-motor neurons. Stimulation of the motor evoked potentials in the lower-limb muscles is reportedly an effective rehabilitation method for restoring muscle strength and function after stroke [8,9].

The H-reflex appears to be effective for investigating orthopedic and physiotherapy development as well as assessing biomechanical aspects [10,11]. The H-reflex, which was first discovered by German physiologist Paul Hoffmann and called the Hoffmann reflex, activates motor neurons through a single synaptic reflex and induces muscle contraction [12]. When electrical stimulation is applied to the peripheral sensory nerves, M-waves that are transmitted directly to the muscles by activation of the motor nerves are initially generated, and then the Ia afferent fibers are excited to generate H-waves [13]. The H-wave has a low threshold and is induced by a weak stimulus. If the intensity of the stimulus is increased, the H-wave disappears and an increase in the M-wave can be seen [14].

The H-reflex has recently been considered the most useful method for diagnosing nerve root pathologies caused by structural pressure, compensating for the shortcomings of medical imaging and electrophysiological examination techniques [9,15]. The H-reflex allows for observation of the excitatory and inhibitory changes in the spinal motor neurons [12]. Electroneurography is performed through the Ia sensory nerve, S1 spinal cord, and α-motor neurons; electric stimulation is administered directly to the peripheral nerves, and the monosynaptic reflex is measured. This method is used to diagnose sacral radiculopathy and quantify spasticity [16].

The H-reflex is commonly used to assess the excitability of the α -motor neuron pool and to offer information at the spinal cord level [17]. The exactness of stimulus delivery, the excitability of the entire H-reflex arch, and the precision of the recording all influence the size strength of the H-reflex [18]. In addition, the roles of the Ia afferent reflex loop have been studied from various aspects, including posture control, single and complex joint motions, and posture stability direction and phase related to the functional task performance of gait [19]. In other words, the H-reflex, an electric stimulation, irradiates the spinal cord motor reflex, which is important for posture control. The H-reflex controls the posture in situations including postural task performance, the motion that requires complex changes, and activity in new angles [20]. The H-reflex amplitudes during the gait cycle were measured using quantitative electromyography (EMG) in both hemiplegic and healthy persons [20]. However, research on the H-wave latency of hemiplegic and normal individuals during the gait cycle is lacking.

Therefore, this study aimed to induce an H-reflex latency in the soleus muscles during walking and compare the influence of individual latencies on the gait cycle in healthy individuals and hemiplegia patients. It also aimed to provide foundational data for the diagnosis and rehabilitation of abnormal muscle activity in hemiplegia patients during H-reflex latency using unilateral absence and bilateral latency as the diagnostic criteria.

## 2. Materials and Methods

### 2.1. Participants and Sampling

A commercially available software application (G*Power version 3.19 statistical power analysis) was used to estimate the sample size based on power calculations and data from similar research on H-reflex latency mean and standard deviation [21]. A needed sample size of 25 was estimated for each group based on a power of 90%, a type 1 error (α) of 5%, a type 2 error (β) of 10%, and a confidence interval of 95% [22,23]. The patient group included individuals being treated at Hospital B for hemiplegia due to cerebral infarction or hemorrhage. The individuals walked comfortably on the treadmill with the EMG pad attached.

The ethical clearance committee for human rights studies gave its approval to the research involving human individuals of Nambu University (1041478-201503-HR-006) and this study was conducted in accordance with the Declaration of Helsinki. Before the intervention began, each participant was given a thorough explanation of the study, and they signed a written informed consent form.

### 2.2. Inclusion and Exclusion Criteria

The selection criteria for the stroke patients were as follows: no visual or vestibular problems caused by stroke and no aphasia, modified Ashworth scale grade 1–2 ankle resistance, and poor to fair ankle dorsiflexion on the manual muscle test. The selection criteria for healthy individuals and hemiplegia patients were as follows: no peripheral nervous or orthopedic disease, capable of linguistic communication and understanding instructions, ability to walk at least 10 m indoors without assistance, and ability to perform the experiment in accordance with the experimenter’s instructions.

The criteria for exclusion from research participation are as follows: those who have joint or muscle problems due to a musculoskeletal disorder, those with severe hypertension or cardiovascular disease diagnosed by a physician, and those who do not understand the contents of the therapy [24]. All subjects met the conditions of not engaging in strenuous exercise for 24 h previous to testing [25] and not consuming meals, stimulants, or smoking for 2 h prior to testing [26].

### 2.3. Measurements

**Figure 1:**
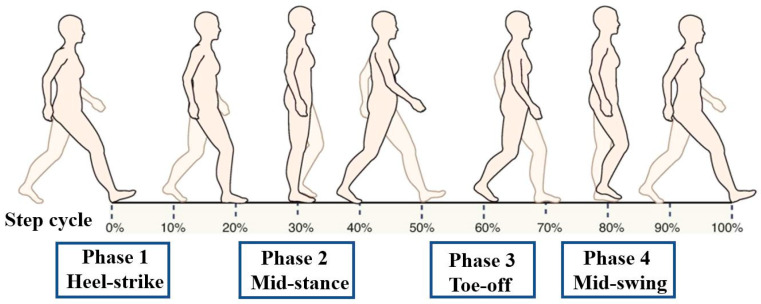
H-reflexes were elicited by stimuli at heel-strike, mid-stance, toe-off, and mid-swing of the stride cycle for all walking conditions.

While the individuals were walking, stimuli were applied to obtain the H-reflex latency changes in four stages: heel-strike, mid-stance, toe-off, and mid-swing (Figure 1). The measurements were carried out by experienced physical therapists with more than five years of expertise. All measures were taken on the right leg, with electrodes inserted on the leg and not withdrawn until the data collection was complete in a setting free of visual and audible distractions [27].

An MP150 (BIOPAC Systems, Inc., Goleta, CA, USA) with Ag-Ag/Cl electrodes (2-cm diameter) was utilized to record and process the EMG signals. The EMG signals were recorded at a sampling rate of 1000 Hz and then processed using full-wave rectification. Data were obtained using Acknowledge 4.1 software (BIOPAC Systems, Inc.) while the participants were walking. The gain was set to 2000 µV; low-pass filtering was set to 10 kHz; high-pass filtering was set to 5 Hz; and the sweep was set to 5 ms. The stimuli were applied in the forward direction at a frequency of 1 Hz, while the stimulation frequency was once every 2 s and the stimulus duration was 1 ms. To measure the H-reflex, band-pass filtering at 30–500 Hz was performed; to remove noise, notch filtering at 60 Hz was performed. After obtaining the waveform for the H-reflex, a maximum stimulus of approximately 1 mV was applied to induce M-waves [12].

A line was drawn between the center of the popliteal fossa folds and the medial malleolus to measure the H-reflex, and a recording electrode was attached to the inner muscle belly of the soleus muscle at the midpoint of the line [12,24]. A standard electrode was attached 15 cm distally from the Achilles tendon. The cathode of the bipolar stimulating electrode utilized in this experiment was positioned proximally on the popliteal fossa to stimulate the tibial nerve. A 1 cm × 1 cm self-adhesive disposable electrode (Neuroline Disposable Neurology Electrodes 700 10-k, Medicotest A/S, Olstykke, Denmark) was attached between the stimulating and recording electrodes for the ground electrode [24,28]. The bipolar electrode was used to administer electrical stimulation at the level of submaximal stimulation at 2-s intervals from the middle of the folds of the hamstring muscle to the posterior tibial nerve. Then, to determine the optimal stimulation intensity for recording H-reflexes and M-waves, the intensity of stimulation was increased from the H-reflex threshold until the M-wave no longer increased [28].

To avoid direct stimulation of the motor neuron axons and M-wave generation, the H-reflex was evoked by low voltage with a current of 1-ms duration at a rate of once per 2 s. It is important to effectively block noise; therefore, the precise analysis location was carefully selected. The electrode was fixed in place with tape to prevent noise due to the electrical wiring [12]. The participants were asked to walk comfortably on the treadmill with the pad attached; the trial began once they had achieved their normal natural gait. The experiment was performed within a range that prevented the individuals from feeling fatigue due to focus and attention. Stimuli were given to the participants while they were walking to obtain an H-reflex at four different stages: heel strike, mid-stance, toe-off, and mid-swing.

During the four gait phases, the H-reflex stimuli were manually administered as a square 0.1-s voltage pulse, with the timing of the stimulation displayed on a screen [28]. By starting with a low-intensity stimulus and increasing it gradually, the H-reflex latency was measured. The latency represents the onset latency measured as the time point at which the waveform began to deviate from the baseline. The maximum amplitude (peak-to-peak) was also measured. For the H-reflex latency measurement, a low-intensity stimulus was first provided, and the intensity was gradually increased to stimulate only the sensory fibers [29].

- Heel strike (HS): The moment of initial contact of the heel with the ground;
- Mid-stance (MS): The phase in which the grounded leg supports the full body weight;
- Toe off (TO): The phase in which the toe lifts off the ground;
- Mid-swing (SW): The phase in which the raised leg passes the grounded leg.

### 2.4. Data Analysis

The data were analyzed using SPSS Statistics 21.0 (IBM Corp., Armonk, NY, USA). The mean and standard deviation (M ± SD) of the physical characteristics of each group and the H-reflex latency data during walking were computed using descriptive statistics. An independent t-test was used to examine mid-swing, heel-strike, mid-stance, and toe-off phase H-reflex latencies during walking between groups. Statistical significance was determined at values of *p* < 0.05.

## 3. Results

### 3.1. General Characteristics of Subjects

This study included two groups of individuals: healthy adults (*n* = 25) and people who had been diagnosed with hemiplegia following a stroke *(n* = 25) (Table 1). During implementation, there were no adverse effects and complications and all participants completed the study. The patient group included 18 men and 7 women assessed at Brunnstrom stage 3–5; the mean duration after stroke onset was 20.88 ± 8.35 months. Five individuals had hemiplegia on the right side, while the other twenty individuals had hemiplegia on the left side. The average National Institutes of Health Stroke Scale score was 9.08 ± 2.57, while the mean Brunnstrom stage score was 3.88 ± 0.78. The healthy group consisted of 15 women and 10 men who did not have any orthopedic conditions that would influence their gait or balance.

### 3.2. The H-Reflex Latencies during Walking

The results for the stroke patients were 29.24 ± 2.07 ms during heel-strike, 26.92 ± 2.33 ms during mid-stance, 26.08 ± 2.70 ms during toe-off, and 27.72 ± 3.09 ms during the mid-swing phase. In contrast, normal adults showed 30.12 ± 3.19 ms during heel-strike, 30.56 ± 3.00 ms during mid-stance, 30.08 ± 2.41 ms during toe-off, and 29.96 ± 3.38 ms during the mid-swing phase (Table 2). In addition, when an independent t-test was used to evaluate the stroke of normal adults, the mid-stance, the toe-off, and the mid-swing showed statistically significant differences in the gait cycle (*p* < 0.05), while there was no significant difference in the heel-strike (*p* > 0.05).

## 4. Discussion

The improvement of gait ability in hemiplegic stroke patients is an essential factor for the functional independence of stroke patients [30]. The joint structure around the disease-related dysfunction reduces the functional independence in proprioception [31]. As a result, problems may arise due to the amount of exercise, movement start time, and anatomical body segment of mobility [20]. Hemiplegic patients show damage to the inhibitory mechanisms, resulting in hypersensitivity of the stretch reflex due to impaired presynaptic inhibition [32]. This interferes with joint exercises during the recovery process, and can cause secondary joint stiffness, leading to inactivity of the dorsiflexor muscles of the ankle joint [33]. However, the interactions between the changes in the spinal reflexes and the improvements in the motor neurons are still poorly understood [34]. Therefore, we emphasize an evidence-based therapeutic approach that analyzes abnormal gait patterns and their causes and objectively evaluates the treatment results.

The H-reflex is a monosynaptic EMG method that is used for the evaluation of the functional states of the reflex loop at the spinal cord level [15]. It reflects the excitability of the α-motor neurons, which are excited via the spinal cord when electrical stimulation is applied to the peripheral nerves and travels up to the afferent fibers from the point of stimulation; it is used as an objective index to measure spasticity.

The H-reflex is important in electrodiagnostic testing because it complements the limitations of other electrodiagnostic testing methods and improves sensitivity and specificity for diagnosing radiculopathies [35]. In patients with ankle spasticity caused by upper motor neuron damage, de-inhibition of the motor neurons leads to H-reflex acceleration and causes abnormal muscle activity during walking. Consequently, the H-reflex is used to assess spinal excitation and to study patterns of altered neural activation and diseases that impair spinal motor neuron excitability [34].

One study on the reliability of the H-reflex in peroneus longus, tibialis anterior, and gastrocnemius reported that the reliability was highest for peroneus longus [36]. In addition, the H-reflex of flexor carpi radialis has been reported to be useful in the differential diagnosis of cervical radiculopathies [37]. However, because the soleus muscle acts on the first sacroiliac joint (SIJ), it can be used broadly for the differential diagnosis of the first SIJ or L5 radiculopathies [17]. Therefore, the soleus H-reflex has been used more diversely as an objective instrument in previous studies to evaluate stiffness after brain injury [15]. As a result, the soleus muscle was selected as the area of interest in this study.

The H-reflex size can vary among individuals depending on skin resistance, subcutaneous fat thickness, and nerve position relative to the stimulus; thus, it has been suggested that the methods should be more objective and quantitative for clinical application [12]. Therefore, in this study, the H-reflex amplitude was excluded from the diagnostic criteria, and the ratio of the H-wave latency on the affected versus the unaffected side was used for the diagnosis instead [28].

A standing posture enhances H-reflex excitability compared to a supine posture. Excitability changes between postures [38]. A standing posture causes more presynaptic inhibition than a supine posture, while presynaptic inhibition of the Ia afferent nerve increases during running versus walking [39]. These inhibitory mechanisms prevent afferent sensory nerve transmissions and motor nerve hyperactivation and play a critical role in the sensorimotor control of posture stability. Therefore, the effects of the spinal motor neurons on excitability are significantly different under loaded weight, which is why the present study measured the changes in H-reflex latency using everyday gait [34].

Previous studies have designated an H-reflex latency change of 1.0–1.62 ms as the diagnostic cut-off value. In assessments of the difference in latency between the affected and unaffected sides, diagnostic cut-off values of 1.0–2.0 ms have been reported. The findings of decreased latency in the affected side relative to the unaffected side were consistent with these results [28].

The H-reflex induced in various muscles is reportedly produced by about 70% of the contraction of the normal protagonists. Reciprocal inhibition is the phenomenon in which the antagonist muscle’s motor neuron activity is actively inhibited during mover activation. The H-reflex is the most obvious antagonist of the inhibitory response by the Ia inhibitory interneuron. The H-reflex has been demonstrated to decrease when spasticity worsens as a result of spinal injury and to increase as functionality is recovered [40]. Foot drop and ankle spasticity are two characteristics that impact walking abilities in post-stroke hemiplegic individuals. Inactivity of the ankle flexor muscles atop the foot makes walking difficult [41]. In the present study, a shortened stance phase and lengthened swing phase were observed on the affected side in stroke patients. This result can be explained by the reduced ankle dorsiflexion that occurs when the calf muscles are stretched in hemiplegic patients during the stance phase of gait [42].

However, the H-reflex varies depending on agonist contraction levels and joint positions. The spastic hypertonia that hemiplegic patients develop is characterized by the overactive H-reflex responses and decreased H-reflex latencies that result from neuromodulation disorders [33]. During the gait cycle, the H-reflex latency was shorter in hemiplegic patients than in healthy individuals in this study. The enhanced excitability of motor neurons resulted in the longest H-reflex latency during mid-stance and the shortest latency during mid-swing in healthy individuals [19]. However, hemiplegic patients showed the longest latency during heel-strike and the shortest latency during toe-off. The onset latency of H-reflex refers to the nerve conduction related to the anterior horn cells of the spinal cord [35]. Gait in adult hemiplegic patients can be characterized as foot drop, equinovarus, genu recuvatum, and stiff-knee gait [43]. There were numerical differences in the heel-strike of H reflex onset latency values in this study, but none were statistically significant differences between the two groups. The most common problem that occurs while walking in stroke patients is dorsiflexion of the ankle joint [44]. The paretic side of stroke survivors usually has a shorter stance phase and a longer swing phase. Furthermore, walking speed is reduced, and stride length is reduced [45].

Nevertheless, one problem with the H-reflex is that it is affected differently by neuronal activity, so it must be used alongside another spasticity scale. This is because an objective and quantitative assessment of spasticity are essential to evaluate functional loss or treatment outcomes and to estimate the prognosis. If we look at the results of the H-reflex latency from the point of view of physical therapy, it is very clinically important to examine the changes in the H-reflex latency in stroke patients with ankle instability [46]. Additionally, since the decrease in excitability on the soleus H-reflex pathway as a result of locomotor training correlates with better walking ability, the study of H-reflex latency is considered to be very meaningful in physiotherapy research [47].

Abnormal gait in hemiplegic patients has been steadily investigated in recent years and, in particular, the use of electromyogram (EMG)-based gait analysis has increased [46]. In our study, we determined the four stages of the walking cycle by collecting EMG data via surface electrodes attached to the lower limb while post-stroke hemiplegic subjects were instructed to walk at their normal comfortable speed on a treadmill. As a result, the present study found that the H-reflex latency for stroke patients’ gait was significantly different during the swing phase, mid-stance, and toe-off. This strategy considers the degree of recovery and the characteristics of patients during gait training for hemiplegic stroke patients. This strategy follows stages of objectification and quantification via a standardized method of nerve conduction related to the anterior horn cell of the spinal cord. Therefore, this study provides appropriate clinical data that take into account various changes and conditions of patients with damage to the central nervous system in addition to providing important data for future hemiplegic gait analysis.

## 5. Conclusions

The H-reflex latency during walking in stroke patients showed a statistically significant difference in swing phase, mid stance, and toe-off in this study. These results can be used as diagnostic data to help account for patient characteristics or measure recovery extent for treatment planning and gait training in hemiplegic individuals for physical therapy. Therefore, this study could provide supporting clinical data for the provision of effective walking strategies and functional training programs in hemiplegic patients.

## Figures and Tables

**Table 1:** General characteristics according to group.

Characteristics	Normal (*n* = 25)	Stroke (*n* = 25)	t	*p* Value
Age (years)	65.52 ± 10.63	67.56 ± 11.25	−0.659	0.513
Height (cm)	165.44 ± 9.36	167.84 ± 9.56	−0.897	0.374
Weight (kg)	65.84 ± 11.02	72.72 ± 9.86	−2.327	0.024 *
Leg length (cm)	80.64 ± 4.36	87.76 ± 5.21	−5.243	0.000 *

* *p* value less than 0.05; data are shown as mean ± standard deviation.

**Table 2:** The H-reflex latencies during walking according to group (ms).

Phase	Group	M ± SD	t	*p* Value
Heel-strike	Stroke	29.24 ± 2.07	−1.157	0.253
	Normal	30.12 ± 3.19		
Mid-stance	Stroke	26.92 ± 2.33	−4.793	0.000 *
	Normal	30.56 ± 3.00		
Toe-off	Stroke	26.08 ± 2.70	−5.515	0.000 *
	Normal	30.08 ± 2.41		
Mid-swing	Stroke	27.72 ± 3.09	−2.444	0.009 *
	Normal	29.96 ± 3.38		

* *p* value less than 0.05; data are shown as mean ± standard deviation.

## Data Availability

Data are contained within the article.
